# Cancer/testis antigens: promising immunotherapy targets for digestive tract cancers

**DOI:** 10.3389/fimmu.2023.1190883

**Published:** 2023-06-16

**Authors:** Huihan Ai, Hang Yang, Liang Li, Jie Ma, Kangdong Liu, Zhi Li

**Affiliations:** ^1^Department of General Surgery, The Affiliated Cancer Hospital of Zhengzhou University & Henan Cancer Hospital, Zhengzhou, Henan, China; ^2^Department of Pathophysiology, School of Basic Medical Sciences, Zhengzhou University, Zhengzhou, Henan, China; ^3^Department of Molecular and Cellular Biology, China-United States (US) Hormel (Henan) Cancer Institute, Zhengzhou, Henan, China; ^4^Research Center of Basic Medicine, Academy of Medical Sciences, Zhengzhou University, Zhengzhou, Henan, China

**Keywords:** cancer/testis antigens, digestive tract cancers, immunotherapy, target, esophagus cancer (adenocarcinoma), gastrointestinal carcinoma

## Abstract

Digestive tract cancers, including esophageal, gastric, and colorectal cancers, are the major cause of death among cancer patients worldwide due to the heterogeneity of cancer cells, which limits the effectiveness of traditional treatment methods. Immunotherapy represents a promising treatment strategy for improving the prognosis of patients with digestive tract cancers. However, the clinical application of this approach is limited by the absence of optimal targets. Cancer/testis antigens are characterized by low or absent expression in normal tissues, but high expression in tumor tissues, making them an attractive target for antitumor immunotherapy. Recent preclinical trials have shown promising results for cancer/testis antigen-targeted immunotherapy in digestive cancer. However, practical problems and difficulties in clinical application remain. This review presents a comprehensive analysis of cancer/testis antigens in digestive tract cancers, covering their expression, function, and potential as an immunotherapy target. Additionally, the current state of cancer/testis antigens in digestive tract cancer immunotherapy is discussed, and we predict that these antigens hold great promise as an avenue for breakthroughs in the treatment of digestive tract cancers.

## 1 Introduction

Cancers affecting the digestive tract, such as esophageal cancer, gastric cancer, and colorectal cancer (CRC), continue to be the primary cause of death among cancer patients worldwide (1). Due to application of endoscopic screening, the detection rate of early-stage digestive tract cancers has increased. However, the mortality is still very high because of the heterogeneity of cancers and little improvement in the standard gold therapy suitable for tumors of the digestive tract. It is therefore essential to search for specific prognostic and predictive molecular signatures to guide targeted, individualized therapy. Immunotherapy, which aims to enhance the body’s natural defenses to eliminate malignant cells, represents a monumental breakthrough in cancer treatment and has revolutionized the field of oncology (2). To develop effective immunotherapy treatments, it is crucial to first identify tumor antigens. Cancer/testis antigens (CTAs) are expressed in the testes and various types of cancer but have limited expression in normal adult somatic cells and tissues. These antigens can be recognized by cytolytic T lymphocytes (CTLs) (3, 4). Moreover, CTAs have been reported to be expressed in digestive tract tumors and exhibit specific biological functions. The upregulation of CTAs has been linked to several unfavorable outcomes commonly associated with cancer (5), including promotion of tumor cell stemness (6, 7), elevation of cancer cell tumorigenicity (8), enhancement of mobility (9) and metastasis (10), and conferment of drug resistance (11). These characteristics render CTAs ideal candidates as novel immunotherapeutic targets in digestive tract cancers. The aim of this review is to highlight the latest advances and hypotheses regarding the involvement of CTAs in the pathogenesis of digestive tract cancers and to investigate their potential as targets for cancer immunotherapy.

## 2 CTAs in the digestive tract cancers

### 2.1 Discovery and types of CTAs

Melanoma antigen-1 (MAGE-1, MAGE-A1, MA2-E), a member of the MAGE family, was the first CTA discovered by Alexander Knuth and Thierry Boon in 1991 (12). With a new method called serological identification of antigens by recombinant expression cloning (SEREX) (13), many more CTAs were uncovered, such as synovial sarcoma, X breakpoint 2 (HOM-MEL-40/SSX2) (13), New York’s esophageal squamous cell carcinoma 1 (CTAG1B, NY-ESO-1) (14, 15), synaptonemal complex protein 1 (SCP1) (16), and CT7 (17). Although the first CTAs was discovered in 1991, the name was defined in 1998 (17). To facilitate the organization of the expanding collection of CTAs, the Cancer-Testis database (CTdatabase, http://www.cta.Incc.br/) was established as a user-friendly interface. Over 730 CTAs belonging to over 100 gene families have been identified in many cancer tissues, where their expression is significantly elevated compared with normal tissues and predominantly restricted to germ cells and trophoblasts. Although not all of them have been demonstrated to induce immune responses, they are collectively referred to as CTAs (18).

Cancer/testis (CT) genes are typically expressed in germ line cells, trophoblasts, and certain cancer cells. CT genes are classified into three groups based on their expression profiles: testis-restricted, testis/brain-restricted, and testis-selective. The majority of CTAs are encoded by CT genes (19). However, due to the lack of a clear and universally applicable definition for CT genes, Oliver Hofmann used multiple *in silico* gene expression analysis technologies to investigate the expression patterns of a set of 153 CTAs in normal and cancer tissues. The CTA genes are further classified into two categories: CT-X and non-X CT genes. CT-X family members are subject to more stringent transcriptional regulation in somatic tissues, making them more suitable for immunotherapy applications (20). The CTdatabase has identified a total of 276 CTA genes, of which 127 (46%) are located on the X chromosome, whereas the remaining are distributed across the autosomes and Y chromosome.

### 2.2 Expression of CTAs in the digestive tract cancers

**Figure 1:**
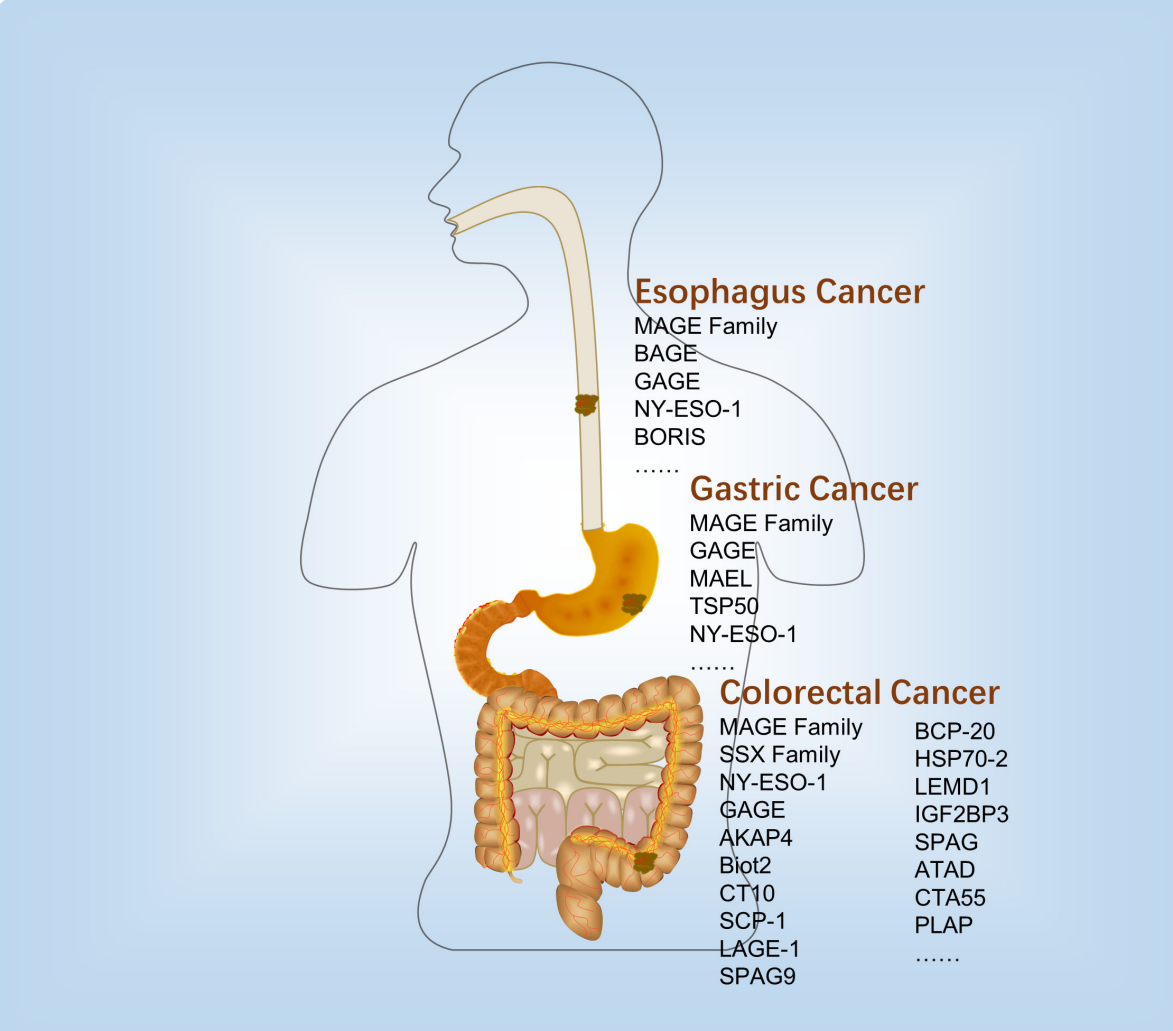
Expression and immunological therapy of CTAs in the digestive tract cancers.

Many CTAs were expressed in the human digestive tract cancers. However, the expression profile was diverse in the different digestive tract cancer tissues and cell lines (**Figure 1** and **Table 1**).

**Table 1:** The expression of CTA in the digestive tract cancers.

Cancer type	Name	Positive/total	Reference
Esophagus cancer	MAGE-1	26/42	21
	MAGE-2	18/42	21
	MAGE-3	24/42	21
	MAGE-A family	38/98	22
	MAGE-A family	111/213	23
	MAGE-A4	38/41	25
	MAGE-A9	57/103	26
	MAGE-A3	11/12	27
	MAGE-A11	37/96	28
	BAGE	Low/48	29
	GAGE	Low/48	29
	GAGE	42/213	23
	NY-ESO	41/123	31
	NY-ESO	44/213	23
	NY-ESO	17/41	25
	BORIS	28/50	33
	LAGE1	16/41	25
Gastric cancer	MAGE-C2	5/51	36
	MAGE-A1	47/86	37
	MAGE-A1	4/41	38
	GAGE	6/51	36
	MAEL	17/80	39
	TSP50	191/334	41
	CT55	3/14	51
Colorectal cancer	MAGE-A family	None/34	42
	MAGE-A2	87/100	45
	MAGE-A7	83/100	45
	MAGE-A8	75/100	45
	MAGE-A12	71/100	45
	MAGE-B2	75/100	45
	MAGE-B3	79/100	45
	MAGE-D2	75/100	45
	MAGE-F1	79/100	45
	MAGE -H1	70/100	45
	MAGE-1	14/121	44
	MAGE-3	33/121	44
	MAGE-4	27/121	44
	CAGE	31/34	42
	LAGE-1	19/121	44
	NY-ESO-1	2/34	42
	NY-ESO-1	12/121	44
	NY-ESO-1	None/62	5
	SSX-1	6/121	44
	SSX-2	3/121	44
	SSX-2	2/34	42
	SSX-4	3/121	44
	SSX-4	3/34	42
	CT-10	8/121	44
	SCP-1	2/121	44
	SPAG9	41/62	5
	AKAP4	27/62	5
	Biot2	108/147	46
	BCP-20	22/57	47
	HSPA2	156/200	48
	LEMD1	17/18	49
	IGF2BP3	56/110	50
	SPAG1	15/110	50
	ATAD	92/110	50
	CTA55	3/18	51
	PLAP	25/116	52

#### 2.2.1 Esophagus cancer

In 1995, Masaki Mori found that MAGE-1, -2, -3 were expressed in 26, 18, and 24 of 42 surgical esophageal cancer tissues and 5, 4, and 4 of 12 human esophageal cancer cell lines, respectively. At least one of the them were expressed in 33 of 42 esophageal tumor tissues, and all of them expressed in 12 of 42 esophageal tumor tissues. However, none of them were expressed in the 42 normal esophageal tissues (21). Apart from that, MAGE-A was detected in 38 of 98 (22) and 111 of 213 (23) esophageal cancer patients.

The MAGE gene family consists of several subfamilies, one of which is the MAGE-A subfamily that includes MAGE-A1 to -A12 (24). In esophageal cancer, several members of the MAGE-A subfamily, including MAGE-A4 (25), MAGE-A9 (26), MAGE-A3 (27), and MAGE-A11 (28) have been detected. In addition to the MAGE-gene family, B melanoma antigen (BAGE) and G antigen (GAGE) families were also expressed in various tumors of different histological origins, including the esophageal squamous and esophageal adenocarcinoma (29). Similarly, they were not expressed in normal tissues other than testis (30).

Additionally, NY-ESO-1, which is also known as cancer/testis antigen 1B (CTAG1), is a prototypical member of the cancer-testis gene family and was originally identified from esophageal cancer (31). NY-ESO-1 is a major CTA in several studies. A study reported that 33% (41 out of 123) of esophageal squamous specimens showed positive mRNA expression for NY-ESO-1 (31). In two other studies, the proportions are 20.7% (23) and 41.4% (25), respectively. Usually, the expression of CTAs was not independent. A reported strong correlation was observed between the expression of cancer/testis antigen 2 (LAGE-1) and the expression of NY-ESO-1 and MAGE genes in esophageal squamous cancer (25, 32). Yutaka Kawakami discovered a new CTA called brother of the regulator of imprinted sites (BORIS), which is expressed in esophageal cancer and may serve as a novel prognostic indicator for patients with this type of cancer (33). BORIS could bind to the promoter of NY-ESO-1 (34) and MAGE-A1 (35) genes to regulate their expression.

#### 2.2.2 Gastric cancer

There is growing evidence showed that several CTAs were expressed in the gastric cancer. The expression of MAGE-C2, also known as CT10, and GAGE was detected in 5 out of 51 and 6 out of 51 gastrointestinal stromal tumor tissues, respectively (36). MAGE-A1 as an important member of MAGE family was detected positive expression in 47/86 (37) and 4/41 (38) gastric cancer tissues. Another CTA, maelstrom spermatogenic transposon silencer (MAEL), was detected in gastric cancer using RT-PCR to measure its mRNA levels. The results indicated that MAEL over- and underexpressions were 17 and 28 out of 80 gastric cancer patients, respectively (39). A 50-kDa serine protease-like protein called testis-specific protease-like protein 50 (TSP50), which is encoded by a CTA gene, was discovered in human breast cancer cells through the isolation of a hypomethylated DNA fragment (40). According to Rongcheng Luo and his colleagues, a study found that the expression of TSP50 was upregulated in a significant proportion of human gastric cancer cases, with 57.2% of samples (191 out of 334) showing overexpression (41).

#### 2.2.3 Colorectal cancer

The expression of the MAGE family in CRC tissues is contradictory. The analysis of 34 CRC samples revealed no expression of the MAGE antigen, specifically MAGE-A1, -A2, -1, -A3, -A12, and -C1 (42). Achim A. Jungbluth and his colleagues detected that MAGE antigens were not expressed in CRC (43). However, it was found that MAGE-1 (11.6%), -3 (27.3%), and -4 (22.3%) were detected to have a positive expression in the CRC tumor samples (44). A different study reported significant overexpression of MAGE-A2 (87%), MAGE-A7 (83%), MAGE-A8 (75%), MAGE-A12 (71%), MAGE-B2 (75%), MAGE-B3 (79%), MAGE-D2 (75%), MAGE-F1 (79%), and MAGE-H1 (70%) in CRC tissues (45). Therefore, more research would be required to better understand the expression pattern of the MAGE family in the CRC. In addition to the MAGE family, other cancer/testis antigens (CTAs) have also been identified in CRC, with NY-ESO-1 being one of the most extensively studied. In a cohort of 121 CRC patients, NY-ESO-1 gene expression was detected. The same study reported that several other CTAs, SSX family gene (10%), CT10 (6.6%), SCP-1 (1.7%), and LAGE-1 (15.7%), were overexpressed in CRC tissues compared with matched adjacent non-cancerous tissues (44). Similarly, the researchers analyzed the CTA levels in 34 CRC tissues and found that two of them were NY-ESO-1 positive. The expressions of SSX-2, SSX-4, and CAGE were respectively 2, 3, and 31 (42). However, a study including 62 Iranian CRC samples was not detected the expression of NY-ESO-1. Approximately 66% and 44% of tumors were observed to express the genes encoding for sperm associated antigen 9 (SPAG9) and a-kinase anchoring protein 4 (AKAP4), respectively (5). Other CTAs expressed in CRC include coiled-coil domain containing 7 (Biot2) (46), F-box protein 39 (BCP-20, FBXO39) (47), heat shock protein family A member 1B (HSP70-2) (48), LEM domain containing 1 (LEMD1) (49), insulin-like growth factor 2 mRNA binding protein 3 (IGF2BP3), sperm-associated antigen 1 (SPAG), acute type A aortic dissection (ATAD) (50), CTA55 (51), and recombinant phospholipase A2 activating protein (PLAP) (52). However, due to the small sample size used in these studies, it is necessary to confirm the results in a larger cohort to validate their findings.

### 2.3 The role of CTAs in the digestive tract cancers

Expressions of CTAs in tumors are perceived as the result of widespread DNA hypomethylation in the carcinogenesis (53). The special expression patterns made them as promising biomarkers and therapeutic targets. There have been numerous clinical research studies and trials conducted to investigate the potential clinical applications of CTAs, but their precise role in cancers is still not well understood.

#### 2.3.1 Prognostic and biomarkers

An increasing body of evidence suggests that CTA expression may have a prognostic role in esophageal, gastric, and colorectal cancers. However, there also a number of CTAs which had no relationship to clinical features of tumors.

##### 2.3.1.1 In esophageal cancer

Studies have found that the presence of MAGE is irrelevant to age, sex, histologic type, depth of wall invasion, lymph-node metastasis, or disease stage (21–23). Additionally, no significant difference was observed between MAGE-A expression and TNM stage, grading, or survival period in patients with the disease (22). A separate study indicated a correlation between tumor progression and the expression levels of MAGE-A4. Specifically, the expression levels of MAGE-A4 were found to be correlated with tumor metastasis to the lymph nodes, and the number of involved lymph nodes was also associated with the level of MAGE-A4 expression (25). Another study found that the expressions of MAGE-A11 (28) and MAGE-A9 (26) in esophageal cancer tissues were significantly correlated with larger tumor size and more advanced tumor stage. Moreover, the expression levels of MAGE-A9 and lymph node metastasis were found to be independent prognostic factors for the overall survival rate of patients with esophageal cancer (26). However, the role of NY-ESO-1 in esophageal cancer is controversial due to conflicting reports on its prognostic value as well as its potential as a target for immunotherapy. One study found that no significant difference was observed in survival rates between NY-ESO-1 protein-positive and -negative cases (31). Nonetheless, co-expression of NY-ESO-1 and MAGE-A4 was significantly correlated with differentiation of esophageal cancer (25). Expressions of MAGE genes have been found to be significantly related to a good prognosis in the absence of BAGE and GAGE expressions. Conversely, the expressions of BAGE or GAGE has been linked to a poor prognosis in cancer patients (29), although there was no significant difference in disease progression, TNM factors, or survival curves with the expression of GAGE (23). BORIS is another biomarker for prognostic diagnosis of esophageal cancer patients. Patients with tumors that tested positive for BORIS had poor overall survival according to one study. Additionally, BORIS expression was identified as an independent poor prognostic factor and was significantly associated with lymph node metastasis (33).

##### 2.3.1.2 In gastric cancer

MAGE-A expression has been linked with lymph node metastasis, poor differentiation, high clinical TNM stage, and inferior patient survival (54). However, MAGE-A expression alone is not deemed an independent prognostic factor in patients with the disease. Conversely, MAGE-A1 expression has been proposed as a predictive marker for resistance to taxane-based chemotherapy in patients with gastric cancer, although it does not directly contribute to drug resistance (38). In high-grade gastrointestinal stromal tumors, MAGE-C2 co-expression with GAGE was significantly correlated with mitotic rate, tumor size, and neoplasm recurrence (36). Additionally, markers for poor relapse-free survival in gastric cancer include MAGE-A1, MAGE-A3, MAGE-A4, MAGE-C1, and NY-ESO-1 (55). High levels of TSP50 were significantly associated with shorter survival time, later TNM stage, and presence of lymph node metastases in patients with the disease. Furthermore, TSP50 overexpression was identified as a significant independent prognostic factor in gastric cancer patients (41). Moreover, in patients with H. pylori-negative gastric cancer, there was a significant correlation between MAEL expression and tumor stage, tumor grade and depth of invasion (39).

##### 2.3.1.3 In CRC

Who exhibited a high protein expression of MAGE-D4 (56) or MAGE-A9 (57), had significantly shorter overall survival compared with those with a low protein expression. Nevertheless, there was no correlation found between MAGE-D4 expression and clinicopathological parameters (56). In patients with colorectal cancer, a high expression of MAGE-A9 was significantly associated with venous invasion, lymph node metastasis, and poor prognostic (57). Similarly, a study conducted in Taiwanese patients with colorectal cancer revealed that MAGE-B3, MAGE-D2, and MAGE-H1 expressions were correlated with tumor size and stage, whereas MAGE-B3 was also correlated with lymph node metastasis (45). In addition, NY-ESO-1 (44) and AKAP4 (5) were found to be significantly correlated with tumor stages and local lymph node metastasis in CRC patients. Biot2 expression was also found to be associated with poor prognosis in early-stage patients with CRC (58).

However, a high expression of CTAs in digestive tract cancers may have prognostic significance or simply exist as a tumor marker without indicating patient prognosis. Differences in detection methods can lead to different rates of CTA detection in various studies, which may result in biased analysis of patient prognosis. Additionally, some patients’ clinical characteristics may be associated with CTA expression, but further research is needed to identify such patients.

#### 2.3.2 Tumorigenesis, development, and metastasis

In addition to being biomarkers for digestive tract cancers, some CTAs also play a key role in the tumorigenesis, development, and metastasis. The absence of BORIS resulted in a decrease of cell proliferation and invasion in the esophageal cancer cell lines (33).Overexpression of MAGE-A1 in the gastric cancer cell lines increased the sensitivity to paclitaxel and docetaxel (38). In a study, SCRN1 was found to be expressed in five of seven gastric cancer patients, and it promoted growth of NIH3T3 cells (59). The knockdown of Biot2 in CRC cell lines has been shown to cause cell cycle arrest in the G1 phase and induce apoptosis by regulating p16 and p21, both *in vitro* and *in vivo (* 60). Furthermore, according to research, ablation of HSP70-2 significantly reduced cellular growth, the colony-forming, migratory, and invasive abilities of CRC cells, and tumor growth of human CRC cell line xenograft (48). Additionally, when researchers screened the transcriptome of cancer stem cells (CSC) of human CRC, they found that LEMD1 was preferentially expressed and its presence was essential for the maintenance of CSC (61). Moreover, according to research, CT55 functions as a stimulator of nuclear factor-κB (NF-κB) signaling induced by tumor necrosis factor (TNF)-α by binding to the IκB kinase complex. Deficiency of CT55 suppresses the development of colitis-associated CRC (62).

CTAs can be used as targets for immunotherapy in digestive tract tumors, allowing immune cells or related immune agents to selectively eliminate tumor cells that express CTAs but minimizing side effects on normal cells. Therefore, the application of CTAs to immunotherapy has become an area of active research and a subject of intense interest. These findings suggest that CTAs represent a promising focus for the treatment of digestive tract cancers.

## 3 CTAs in the immunotherapy of digestive tract tumor therapy

**Figure 2:**
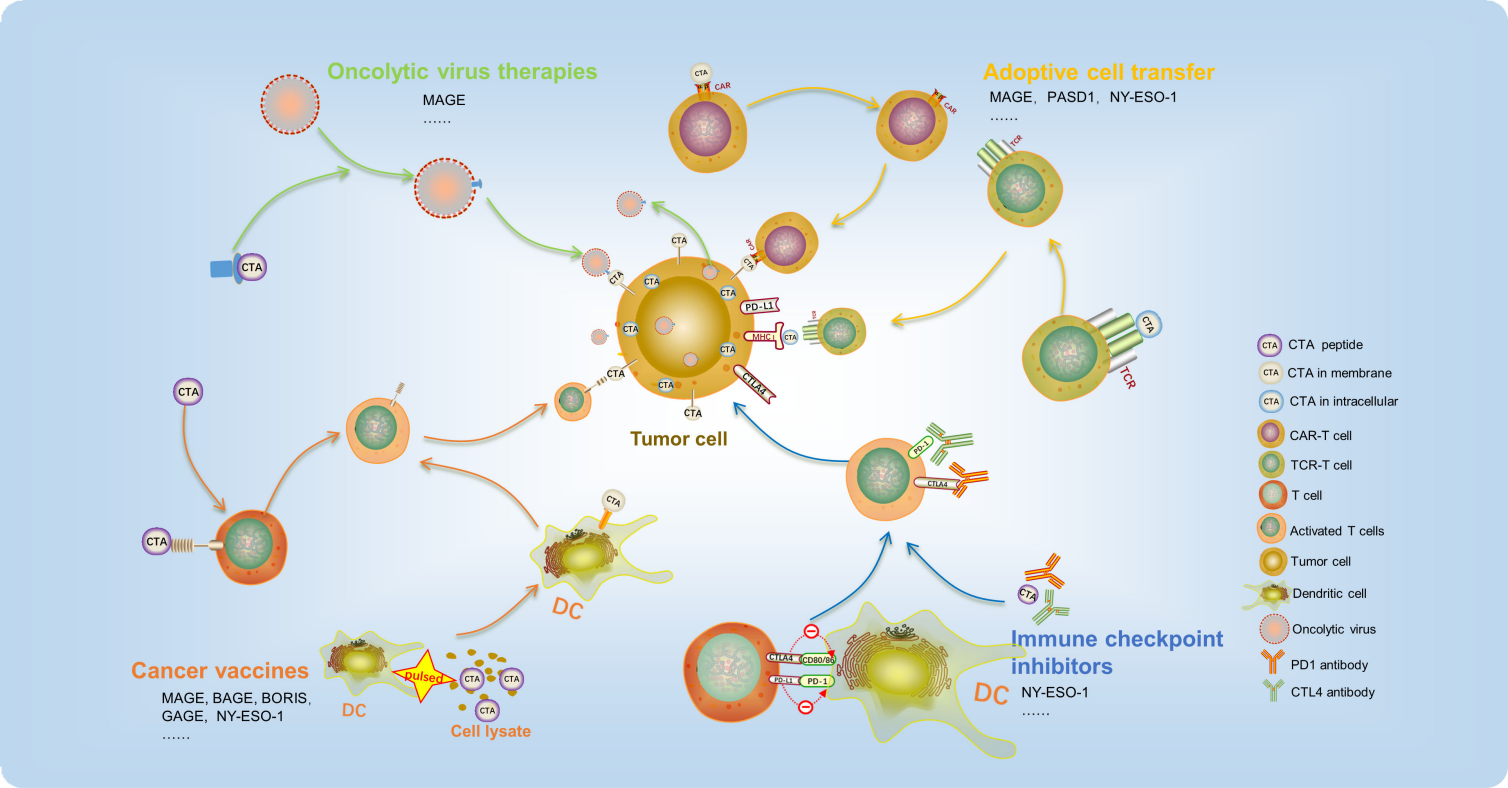
CTA-based immunotherapy has recently been used in cancer treatment and achieved promising outcomes. It mainly includes oncolytic virus therapies, cancer vaccines, adoptive cell transfer, and immune checkpoint inhibitors.

During the course of cancer development, tumor antigens can be identified as aggressor by the immune system, which triggers cellular immune responses. While T-cell-related immunotherapy has received significant attention, research has also demonstrated that other immune cells of both the innate and adaptive immune systems, such as DCs, macrophages, NK cells, and B cells, play a crucial role in facilitating immunotherapy responses. In cancer treatment, the major types of immunotherapies applied are oncolytic virus therapies, cancer vaccines, adoptive cell transfer (ACT), and immune checkpoint inhibitors (ICIs). Due to the limited expression of CTAs in tumors, their high immunogenicity, and their biased expression, CTA-based immunotherapy has emerged as a promising approach in cancer treatment, showing encouraging results in preclinical and early clinical trials (**Figure 2**).

During cancer development, tumor antigens can be recognized as aggressors by the immune system, triggering cellular immune responses. While T-cell-related immunotherapy has received significant attention, research has demonstrated that other immune cells of both the innate and adaptive immune systems, such as DCs, macrophages, NK cells, and B cells, play a crucial role in facilitating immunotherapy responses. Types of CTA-based immunotherapy applied in digestive tract tumors treatment include oncolytic virus therapies, cancer vaccines, adoptive cell transfer (ACT), and immune checkpoint inhibitors (ICIs). Due to their limited tumor expression, high immunogenicity, and biased expression, CTAs have emerged as a promising strategy in cancer treatment (**Table 2**), showing encouraging results in preclinical and early clinical trials.

**Table 2:** The application of CTA in clinical trials.

Clinical trial number	Number of patients	Types of cancer	Phase	Status	Treatment types	Target	Study year	Reference
NCT02285816	56	Esophagus cancer, gastric cancer	1/2	Active, not recruiting	OVs (MG1MA3, AdMA3)	MAGE-A3	2014-2019	64, 65
NCT00020267	26-56	Colorectal cancer	1	Completed	Peptide vaccine	MAGE-12	2007-2015	Not provided
NCT05130060	15	Colorectal cancer	1	Active, not recruiting	Peptide vaccine (PolyPEPI1018)	Multiple CTAs	2022-2023	72
NCT05243862	28	Colorectal cancer	2	recruiting	Peptide vaccine/ICIs (PolyPEPI1018)	Multiple CTAs	2022-2024	Not provided
NCT01003808	25	Esophagus cancer	1	Completed	Protein vaccine (IMF-001)	NY-ESO-1	2009-2012	70
NCT01522820	18	Esophagus cancer, gastric cancer, colorectal cancer	1	Completed	Protein vaccine (CDX-1401)	NY-ESO-1	2012-2016	Not provided
NCT00291473	9	Esophagus cancer, gastric cancer	1	Completed	Protein vaccine (CHP-NY-ESO-1)	NY-ESO-1	2005-2008	Not provided
NCT00199849	18	Esophagus cancer	1	Completed	Plasmid DNA (pPJV7611)	NY-ESO-1	2004-2006	Not provided
NCT01234012	23	Esophagus cancer	1	Completed	Protein vaccine (IMF-001)	NY-ESO-1	2011-2013	Not provided
NCT00948961	70	Colorectal cancer	1/2	Completed	Protein vaccine (CDX-1401)	NY-ESO-1	2009-2012	71
NCT00106158	9	Esophagus cancer	1	Completed	Protein vaccine	NY-ESO-1	2004-2006	69
NCT00682227	10	Esophagus cancer	1	Unknown	Protein Vaccine	TTK, LY6K, IMP-3	2006-2008	73
NCT00311272	40	Colorectal cancer	2	Completed	Protein vaccine (MelCancerVac)	MAGE	2004-2007	76, 77
NCT05430555	48	Esophagus cancer, gastric cancer	1/2	Recruiting	TCR-T (TK-8001)	MAGE-A1	2022-2024	Not provided
NCT03132922	52	Esophagus cancer, gastric cancer	1	Active, not recruiting	TCR-T (MAGE-A4c1032T)	MAGE-A4	2017-2032	89
NCT04752358	45	Esophagus cancer, gastric cancer	2	Active, not recruiting	TCR-T (ADP-A2M4CD8)	MAGE-A4	2021-2023	92
NCT04044859	120	Esophagus cancer, gastric cancer	1	recruiting	TCR-T(ADP-A2M4CD8)	MAGE-A4	2019-2023	91
NCT02096614	18	Esophagus cancer	1	Completed	TCR-T (TBI-1201)	MAGE-A4	2014-2021	Not provided
UMIN000002395	9	Esophagus cancer	Unknown	Completed	TCR-T	MAG4	2009	88
NCT01795976	2	Esophagus cancer	2	Terminated	TCR-T	NY-ESO-1	2014-2017	Not provided
NCT03159585	6	Esophagus cancer, gastric cancer	1	Completed	TCR-T (TAEST16001)	NY-ESO-1	2017-2019	Not provided
NCT02869217	22	Esophagus cancer	1	Active, not recruiting	TCR-T(TBI-1301)	NY-ESO-1	2016-1013	Not provided
NCT05483491	42	Gastric cancer	1	Recruiting	TCR-T	KK-LC-1	2022-2023	Not provided
NCT05035407	100	Gastric cancer	1	Recruiting	TCR-T	KK-LC-1	2022-2025	Not provided
NCT00037817	34	Esophagus cancer	1	Completed	DAC	MY-ESO-1	2002-2008	Not provided
NCT00623831	34	Esophagus cancer	1	Completed	Mixed bacteria vaccine	NY-ESO-1	2007-2013	Not provided

### 3.1 Oncolytic virus therapies

Oncolytic viruses (OVs) are a unique category of viruses that selectively infect and destroy tumor cells while leaving normal cells unharmed, thanks to their exceptional oncolytic activity and targeting ability. Once inside the tumor cells, OVs can multiply and release new viral particles, which then infect other nearby tumor cells. Additionally, OVs stimulate an antitumor immune response at the local or systemic level, modify the tumor microenvironment, and amplify their antitumor effects. Currently, five types of oncolytic viruses have been approved for clinical use, and many other preclinical studies are underway (63). The application of CTA in oncolytic virus therapy is also currently under investigation. Ad-MAGEA3 (AdMA3) is a replication-deficient adenovirus (E1/E3-deleted) of human serotype 5 that carries a transgene encoding human MAGE-A3 gene. MG1-MAGEA3 (MG1MA3) is an oncolytic rhabdovirus Maraba with replication competency, created by introducing the human MAGE-A3 transgene between the G and L genes of the attenuated MG1 strain. Jonathan G. Pol confirmed the safety of the Ad : MG1 oncolytic vaccination approach in non-human primates (64). Moreover, they initiated clinical trials for solid tumor treatment, including esophageal cancer and gastric cancer (NCT02285816). The Ad : MG1 oncolytic virus has the ability to replicate within the bloodstream and activate an adaptive, antitumor cellular response in cancer patients. In three out of six evaluated patients, antitumor immunity was observed, with over 1% of total circulating CD8+ T cells reacting against MAGE-A3 in one participant (65). This strategy that modified oncolytic viruses with CTAs as target could eliminate the tumor cells specifically and provides an immunotherapy tool for future digestive tract tumor therapy clinical application.

### 3.2 Cancer vaccines

Cancer vaccines are utilized to deliver tumor antigens into antigen-presenting cells and stimulate T-cell-mediated antitumor immune responses. Vaccines made from a peptide expressed specifically in the tumor may induce the tumor immune response. In patients with digestive tract cancer, specific T-cell responses can be induced by immunogenic epitopes derived from CTAs such as MAGE, BAGE, GAGE, and NY-ESO-1 (66, 67). Up to now, there has been some clinical trial focused on targeting MAGE and NY-ESO-1 that have employed peptide vaccines as a treatment option for digestive tract tumor. (**Table 2**). Three peptide vaccines, CHP-NY-ESO-1, IMF-001, and CDX-1401, have been constructed targeting NY-ESO-1. CHP-NY-ESO-1 is a recombinant protein that consists of NY-ESO-1 and a polysaccharide-based delivery system. The safety of this peptide vaccine has been demonstrated through *in vitro* and animal experiments, indicating their potential for use in clinical trials (68). A clinical trial (NCT00106158) was conducted using CHP-NY-ESO-1 vaccine for 13 patients with advanced esophageal cancer. The study observed the induction of CHP-NY-ESO-1 immunity and some favorable clinical outcomes in patients, without any major toxicities or adverse events (69). Results from other clinical trials (NCT01003808) have demonstrated that CHP-NY-ESO-1 can trigger an immune response in patients with esophageal cancer, leading to a reduction in tumor size. The degree of reduction was observed to increase with increasing dosage (70). CDX-1401 is a vaccine that consists of a human monoclonal antibody specific for DEC-205 fused to the full-length tumor antigen NY-ESO-1. CDX-1401 has the capacity to deliver NY-ESO-1 to DCs through DEC-205 and augment the body’s immune response. Clinical trial (NCT00948961) results have demonstrated that two out of four patients with colorectal cancer experienced stabilized conditions after treatment (71).

Additionally, PolyPEPI1018 is a readily available, multipeptide vaccine consisting of 12 immunogenic epitopes derived from seven cancer testis antigens (CTAs) that are frequently expressed in patients with colorectal cancer. In clinical trials of metastatic colorectal cancer, PolyPEPI1018 was found to elicit an immune response and T-cell infiltration in MSS-type patients. In comparison with TAS-102 alone, the combination of PolyPEPI1018 plus TAS-102 has demonstrated good tolerability, and it can elicit immune responses in peripheral blood and tumor tissue of patients with a lower likelihood of causing grade 3 adverse events (NCT05130060) (72). A phase I clinical trial (NCT00682227) was conducted to examine the safety, immunogenicity, and antitumor effect of a cancer vaccine targeting TTK protein kinase, lymphocyte antigen 6 family member K (LY6K), and insulin-like growth factor 2 mRNA binding protein 3 (IMP-3) against esophageal squamous cell carcinoma. 50% of the 10 enrolled patients showed favorable clinical responses after receiving the vaccination (73). SCRN1 is another CTA identified in gastric cancer tumor tissue. The CTL clones stimulated by SCRN1 were able to recognize tumor cells that expressed the natural SCRN1 protein endogenously (59).

DCs are considered the most efficient antigen-presenting cells and play critical roles in eliciting antitumor immunity (74). In addition to serving as tumor antigens, CTAs have also been utilized in the development of vaccines delivered by dendritic cells (DCs), which have demonstrated significant clinical outcomes. After 4T1 mammary tumor implantation, mice that were vaccinated with a BORIS-based DC vaccine showed a robust anticancer immune response. The tumor growth was inhibited, and the number of spontaneous clonogenic metastases was also lowered significantly (75). In addition, the effect of DC vaccine on patients with advanced CRC were evaluated. The process of generating MAGE-DCs involves pulsing autologous peripheral blood mononuclear cells with allogeneic tumor cell lysate that contains high levels of MAGE (NCT00311272). The MAGE-DCs can present MAGE antigen to T cells and stimulate an antitumor immune response (76). Moreover, the MAGE-DCs were safe and non-toxic. After treatment with the MAGE-DC vaccine, 24% (4/17) of the patients showed stable disease (77). Taken together, these findings provide compelling evidence for the potential utility of CTAs as vaccines in immunotherapy for digestive tract tumors.

### 3.3 ICIs

Immune checkpoints are molecules involved in co-inhibitory signaling pathways that help maintain immune tolerance. However, cancer cells often hijack these pathways to evade immunosurveillance (78). To counteract this, ICIs such as programmed cell death 1 (PD-1), programmed cell death 1 ligand 1 (PD-L1), and cytotoxic T lymphocyte-associated antigen-4 (CTLA-4) antibodies have been developed. These drugs aim to reactivate antitumor immune responses by blocking coinhibitory signaling pathways and promoting immune-mediated elimination of cancer cells. However, remarkable efficacy has been observed with ICI only in a subset of patients. The most widely used methods for ICIs was combination with other chemicals to treat cancers. Similarly, combination treatment with ICIs and CTAs can enhance the body’s immune response. McAuliffe et al. developed a vaccine consisting of a chimpanzee adenovirus (ChAdOx1) and a modified vaccinia Ankara (MVA) that encodes MAGE-type antigens. In murine tumor models expressing P1A, the combination of ChAdOx1/MVA with anti-PD-1 antibody produced superior tumor clearance and survival when compared with treatment with anti-PD-1 alone (79). Thus far, favorable outcomes have been observed in other types of tumors through the utilization of a combination of CTAs and ICIs (80). Clinical trials are currently underway to investigate the combined treatment of PolyPEPI1018 and atezolizumab for colorectal cancer, and results are pending (NCT05243862). Thus, CTA antibodies are also potentially biomarkers predicting and monitoring response to ICI therapy.

### 3.4 ACT

ACT therapies refer to the use of autologous immune cells, mainly T cells, that are extracted, modified, and reinfused into patients to target and eliminate cancer cells. These therapies have demonstrated long-lasting clinical efficacy. There are two types of ACT therapies, namely, chimeric antigen receptor-modified T-cell (CAR-T) immunotherapy and T-cell receptor T cell (TCR-T) immunotherapy (81, 82).

While CAR T-cell therapy has demonstrated impressive outcomes in certain types of B-cell cancers, its applicability to other malignancies, including solid tumors is impeded by the absence of appropriate surface antigens (83). An example of the successful application of CAR T-cell therapy in solid tumors is the MAGE-A1-specific CAR, which demonstrated cytotoxic activity *in vitro* and *in vivo*. It was able to infiltrate tumors that express MAGEA1 and specifically inhibit the growth of lung adenocarcinoma xenografts in nude mice (84). Furthermore, PAS domain-containing repressor 1 (PASD1) is another CTA that has been found to be immunogenic in CRC samples. CD8^+^ T cells, induced by the PASD1 peptide, were shown to be capable of killing HLA-A*24:02^+^ PASD1^+^ cells (85). The researchers, led by Vita Golubovskaya, utilized a single-chain Fv fragment from a mouse monoclonal antibody clone specific to alkaline phosphatase, placental (PLAP), to engineer PLAP-CAR-T cells. These humanized PLAP-CAR-T cells were then shown to significantly inhibit tumor growth in a colon cancer xenograft model (52). However, the expression of CTAs is mainly intracellular, which limits their potential as targets for CAR therapy.

Major histocompatibility complexes (MHCs) present intracellular antigens associated with tumors, which can be targeted by T-cell receptors (TCRs). One type of antigenic target for TCR T cells are cancer-testis antigens (86). The growth of MAGE-A4-expressing esophageal cancer was hindered in NOG mice through the use of genetically engineered T cells that expressed a MAGE-A4-specific TCR designed to target the MAGE-A4 143-151 peptide-NYKRCFPVI, which is restricted to HLA-A24 (87). Furthermore, the use of MAGE-A4-specific TCR in adoptive immunotherapy for patients with recurrent esophageal cancer has been reported as safe (UMIN000002395) (88). In a phase I clinical trial (NCT03132922), Hong et al. evaluated the safety, clinical activity, and translational effects of MAGE-A4-specific TCR (89)in the treatment of solid tumors such as gastric cancer and esophageal cancer. All 38 patients across nine different tumor types experienced grade ≥3 hematologic toxicities; cytokine release syndrome was reported in 55% of patients, with 90% of these being grade ≤2. The objective response rate (ORR) (all partial response) was 24% (9/38). Phase II clinical trials are currently enrolling participants (NCT04044768) (90). In the phase I SURPASS trial (NCT04044859), the safety and efficacy of next-generation ADP-A2M4CD8 SPEAR T-cells that co-express the CD8a coreceptor with an engineered TCR targeting MAGE-A4 were evaluated. In this study which included 18 patients (two with esophageal cancer and four with gastric cancer), the results indicated that the TCR-T cells were safe for use within the human body. The best overall responses observed in the study were one partial response (gastric cancer), four cases of stable disease (two of which were gastric cancer and two were esophageal), and one case of progressive disease (gastric cancer) (91). The phase II clinical trial (NCT04752358) of this TCR-T-cell therapy in esophageal and gastric cancer is currently ongoing, and preliminary results suggest that the clinical outcomes are promising (92).

Furthermore, additional clinical trials have assessed the safety, tolerability, and efficacy of NY-ESO-1 and KK-LC-1-specific TCR gene-transduced T lymphocytes in treating tumors of the digestive tract (**Table 2**). However, as with the prior studies, only one trial has been completed thus far, and its results are pending publication. These findings form the foundation for future clinical investigations aimed at targeting CTAs with ACT therapies in digestive tract tumors.

## 4 Opportunity and challenge

**Figure 3:**
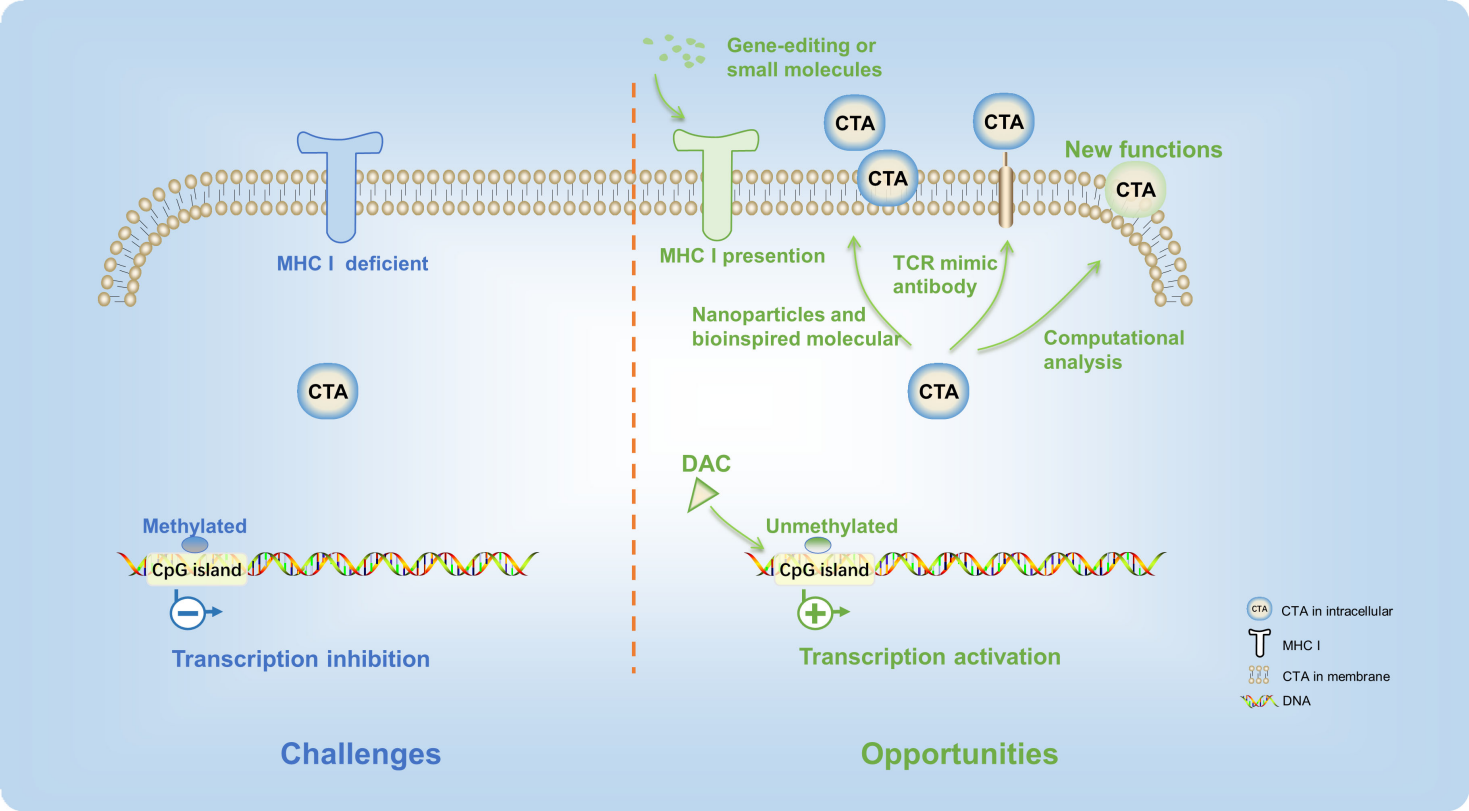
There are many detours and challenges in the CTA-based digestive tract tumor immunotherapy. New strategies and cutting-edge technology provided opportunities to overcome these difficulties.

Currently, surgery, radiation, and chemotherapy remain the major treatment means of patients with digestive tract tumors. The immune therapeutics have not been used as the first line of digestive tract tumor therapy in the clinical setting. Although recent progresses in cancer immunotherapy therapies have been very rapid, their efficacy is still limited to a very small subset of cancer patients. While CTA-based immunotherapies show great potential, the full therapeutic benefits of CTA-targeted digestive tract tumors have yet to be fully realized. There are also many detours and challenges along the way. To overcome the barriers and increase the efficacy of CTA-targeted digestive tract tumor immunotherapy, new strategies and cutting-edge technology should be applied (**Figure 3**).

First of all, since the majority of CTA protein targets are intracellular, tumor cells are often not recognized by specific antibodies or active immune cells, thus presenting a major challenge in CTA-targeted therapies (93, 94). Consequently, most CTAs are unable to elicit a robust immune response in cancer patients. With the defect of cytoplasmic localization, TCR mimic antibodies present new opportunities for additional CAR strategies targeting CTAs (82). TCR mimic antibodies have specificities that resemble those of T-cell receptors, targeting peptides presented in complex with MHC or HLA-I (95). This method enabled HLA-A2/NY-ESO-1 peptide-specific CARs to recognize tumors, offering a promising avenue to expand the range of CAR T-cell targets (96). Likewise, there has been considerable interest in bispecific antibody-based therapeutics that aim to target intracellular oncoproteins (97). This approach expands the range of CTAs that can be targeted and enhances the effectiveness of conventional antibody-based therapeutics. Meanwhile, screening more CTAs located in the membrane of digestive tract cancer cells is an alternative approach. PRAME, a CTA, was previously recognized as an intracellular protein. In recent years, a computational analysis of transmembrane proteins has predicted that a particular protein has an extracellular region that could be targeted specifically by PRAME-specific antibodies *in vitro* and *in vivo (* 98). Therefore, the advances of science and technology could help to find new functions of existing CTAs.

After that, in cancer cells, the MHC-I protein is usually deficient (99), leading to low amounts of CTA epitopes on the cell membrane surface. Therefore, the T cells could not capture tumor antigen. Promoting the transcription of the MHC gene through gene-editing technology or stimulation of small molecules is an effective strategy to improve the efficacy of CTA presentation. Advanced biomaterials, such as nanoparticles and bioinspired molecular (100), could also effectively harness immunotherapies of CTA and improve their potency.

Finally, the inconsistent expression level of CTAs in the digestive tract cancer patients limited their clinical application. Although many CTAs were expressed in digestive tract cancers, only few of CTA-targeted immune therapeutics exhibited high anticancer efficacy. One of the main reasons is that the expression level of CTAs was inhibited by the high DNA methylation level at the promoter regions (101). On account of this, the demethylation agent, such as decitabine (5-aza-2′-deoxycytidine, DAC), was applied to improve antigen-specific T-cell immune responses (102). Expressions of MAGE-A (27, 103), MAGE-3, NY-ESO-1 (104), beta-2-microglobulin, calreticulin, CD58, proteasome 20S subunit beta 8 (PSMB8), and PSMB9 (105) were increased significantly in esophageal cancer and CRC after the treatment of decitabine. Moreover, clinical studies are currently underway to investigate the regulation of CTA expression by DAC (NCT00037817). Furthermore, research has also demonstrated that a mixed bacterial vaccine can activate the body’s immune response and serve as an immune modulator, thereby promoting the combination of NY-ESO-1-positive tumor cells with antigen-specific cancer vaccines (NCT00623831) (106). Reports suggest that therapeutic interventions such as radiotherapy may enhance the release of the NY-ESO-1 antigen from the tumor, which could play a critical role in directing tumor immunotherapy (107, 108). Moreover, the overexpression of CTA in the tumor cells not only improved the antitumor efficacy of T cells but also increased sensitivity of tumor cells for immunotherapy in the digestive tract tumors (109). These approaches have the potential to modulate the extent and phenotype of the antitumor immune response, thus increasing the efficacy of CTA-targeted immunotherapy for digestive tract tumors.

In summary, CTA-based immunotherapies provided a new platform and opportunity for the development of therapeutics for digestive tract tumors. It is anticipated that these novel strategies and approaches will bring about significant breakthroughs in the field of digestive tract tumors immunotherapy in the near future.

## Author contributions

HA: Conceptualization, Investigation, Writing - review & editing, Writing - original draft. HY: Conceptualization, Investigation, Data curation, Writing - original draft. LL: Conceptualization, Investigation, Data curation, Methodology. JM: Conceptualization, Investigation, Methodology. KL: Conceptualization, Investigation, Funding acquisition. ZL: Conceptualization, Investigation, Methodology, Writing - review & editing. All authors contributed to the article and approved the submitted version.
